# Hospital-treated infectious diseases and the risk of epilepsy in older age

**DOI:** 10.1038/s43587-025-01005-x

**Published:** 2025-11-04

**Authors:** Qiyuan Zhuang, Yihan Hu, Dang Wei, Chenxi Qin, Kejia Hu, Junyi Zhang, Lida Chen, Zhelun Yang, Weimin Ye, Karin Wirdefeldt, Xiang Zou, Ying Mao, Sara Hägg, Fang Fang

**Affiliations:** 1 Department of Neurosurgery, Huashan Hospital, Fudan University, Shanghai, P. R. China; 2 National Center for Neurological Disorders, Huashan Hospital, Fudan University, Shanghai, P. R. China; 3 Unit of Integrative Epidemiology, Institute of Environmental Medicine, Karolinska Institutet, Stockholm, Sweden; 4 Department of Medical Epidemiology and Biostatistics, Karolinska Institutet, Stockholm, Sweden; 5 Corporate & Investment Bank Technology, JPMorgan Chase, Houston, TX USA; 6 Hainan Medical University, Hainan, P. R. China; 7 Department of Clinical Neuroscience, Karolinska Institutet, Stockholm, Sweden

**Keywords:** Epilepsy, Infectious diseases, Ageing

## Abstract

Infectious diseases are known to trigger acute seizures, but their long-term impact on epilepsy, especially in later life, is unclear. We conducted nested case–control studies of newly diagnosed epilepsy after age 50 in the UK Biobank (2,486 cases; 12,430 controls) and Swedish registers (56,266 cases; 281,330 controls), including a sibling comparison. Previous hospital-treated infections were associated with a persistently elevated epilepsy risk (for example, >10 years after infection: odds ratio (OR) 1.68, 95% confidence interval: 1.39–2.04 in UK Biobank; 1.46, 1.41–1.51 in Sweden). Associations were robust in sibling analyses and across infection types and sites. We further found that infections, together with a high cardiovascular genetic risk (OR 2.62, 2.22–3.08), a high cardiovascular risk score (OR 3.14, 2.68–3.68) or cardiovascular disease history (OR 4.77, 4.64–4.91), were associated with the highest epilepsy risk. Hospital-treated infections exert prolonged impact on epilepsy risk in older age, especially when in combination with cardiovascular risk factors.

## Abstract (web summary)

Infectious diseases can cause acute seizures. Here the authors investigate their long-term impact on the risk of epilepsy for older adults. They find that hospital-treated infectious diseases have a lasting impact on the development of epilepsy in older age, and that cardiovascular risk factors may exacerbate this impact.

## Main

Epilepsy encompasses a group of neurological disorders characterized by recurring unprovoked epileptic seizures^1,2^, and it manifests both during childhood and in older age^3^. The incidence of epilepsy in older age sharply rises after age 50 and is the highest among individuals at age 75 or above^3–5^. Despite this, there is no universally accepted definition for late-onset epilepsy^6^. Diagnosing epilepsy in older adults is challenging due to incomplete patient history, atypical symptoms, presence of comorbidities, and so on^7,8^.

Infectious diseases have long been recognized as important risk factors for epilepsy^9^. In developed countries, approximately 7–8% of patients recovering from brain infections develop seizures; the risk is even higher in developing countries^10–12^. These acute symptomatic seizures can evolve into a diagnosis of epilepsy and are often associated with lesions visible by magnetic resonance imaging, indicating a direct and immediate impact of cerebral infections. The influence of infectious diseases on epilepsy development may extend beyond the immediate aftermath. A Swedish study of individuals over age 18 revealed an elevated 10-year risk of epilepsy following brain infections^13^, whereas a Danish study found that patients with brain abscesses demonstrated an increased risk of new-onset epilepsy for up to a decade after the initial infection^14^. Recent advances in the understanding of interorgan communications suggest that infections may be linked to neurological disorders through the nose–brain, lung–brain and gut–brain axes^15^. Consequently, it is plausible that infections not directly affecting the central nervous system (CNS) might also modulate the risk of epilepsy. In another Danish study, pertussis was for instance found to be associated with an increased long-term risk of epilepsy in children^16^. Nonetheless, real-world evidence on the impact of infections on late-onset epilepsy remains insufficient.

Beyond infectious diseases, various genetic and nongenetic factors have been proposed for epilepsy^3^. The largest genome-wide association studies, including 15,212 individuals with epilepsy, identified numerous loci with diverse functions, including genes coding for ion-channel subunits, vitamin B_6_ metabolism and transcription factors^17^. Cardiovascular risk factors are among the most discussed risk factors for epilepsy^18^. For instance, it is estimated that approximately 9% of unprovoked seizures are attributed to cerebrovascular disease^19^. Studies have further demonstrated a robust association between different vascular risk factors and risk of epilepsy, independently of stroke^20,21^. Despite these insights, little is known about potential joint effects between infectious diseases and other risk factors of epilepsy. A synergy is plausible as mechanistic studies have suggested that the prolonged effects of infectious diseases on susceptibility to epilepsy may be via the neuro–immune–cardiovascular axis^22,23^.

To address these knowledge gaps, we conducted a comprehensive analysis using data from the UK Biobank and Swedish registers, to investigate the association between previous hospital-treated infectious diseases and risk of epilepsy in older age. We performed two nested case–control studies to (1) assess the temporal impact of infectious diseases on the risk of epilepsy above age 50; (2) evaluate the impact of infectious diseases from different anatomical sites or of varying types; and (3) explore the joint effects between infectious diseases and other risk factors (that is, genetic susceptibility to epilepsy and cardiovascular risk factors). Finally, to alleviate potential concern of familial confounding, we also performed a sibling analysis using data from the Swedish registers.

The incidence rate of epilepsy was 69.0 per 100,000 person-years in the UK Biobank cohort, and 138.3 per 100,000 person-years in the cohort of Swedish registers. In the UK Biobank, we included 2,486 epilepsy cases alongside 12,430 matched controls, with the mean age at the index date (that is, date of diagnosis for the case and their individually matched controls) as 65.5 years (Table 1). In the Swedish registers, we included 56,266 epilepsy cases alongside 281,330 matched controls, with the mean age at the index date as 70.1 years. In the sibling analysis, the mean age at the index date was 63.1 for epilepsy cases and 62.5 years for the unaffected siblings. Compared to controls, individuals with epilepsy generally had relatively lower levels of education and annual household income.

**Table 1:** Characteristics of the cases and controls included in the analysis of the UK Biobank and Swedish registers

UK Biobank			Swedish registers				
Characteristics	Cases (*n* = 2,486)	Matched population controls (*n* = 12,430)	Characteristics	Cases (*n* = 56,266)	Matched population controls (*n* = 281,330)	Sibling comparison	
						Cases (*n* = 29,044)	Sibling controls (*n* = 63,447)
Age, mean (s.d.)	66.5 (7.6)	66.5 (7.6)	Age, mean (SD)	70.1 (12.3)	70.1 (12.3)	63.1 (8.6)	62.5 (9.3)
Sex, *n* (%)			Sex, *n* (%)				
Female	1,170 (47.1)	7,020 (47.1)	Female	25,735 (45.7)	128,675 (45.7)	12,332 (42.5)	31,172 (49.1)
Male	1,316 (52.9)	7,896 (52.9)	Male	30,531 (54.3)	152,655 (54.3)	16,712 (57.5)	32,275 (50.9)
Educational attainment, *n* (%)			Educational attainment, *n* (%)				
College or university degree	631 (25.4)	4,458 (29.9)	>12 years	5,762 (10.2)	37,791 (13.4)	3,400 (11.7)	8,031 (12.7)
A levels/AS levels or equivalent	217 (8.7)	1,542 (10.3)	9–12 years	26,123 (46.4)	136,175 (48.4)	15,247 (52.5)	34,449 (54.3)
O levels/GCSEs or equivalent	488 (19.6)	2,961 (19.9)	<9 years	23,118 (41.1)	100,584 (35.8)	9,691 (33.4)	19,340 (30.5)
Certificate of Secondary Education or equivalent	123 (4.9)	612 (4.1)	Unknown	1,263 (2.2)	6,780 (2.4)	706 (2.4)	1,627 (2.6)
National Vocation Qualification or HND or HNC or equivalent	173 (7.0)	978 (6.6)					
Other professional qualifications	135 (5.4)	828 (5.6)					
Unknown	719 (28.9)	3,537 (23.7)					
Annual household income in £, *n* (%)			Annual household income, *n* (%)				
<18,000	674 (27.1)	3,374 (22.6)	Lowest 20%	14,264 (25.4)	51,883 (18.4)	7,510 (25.9)	9,464 (14.9)
18,000–30,999	565 (22.7)	3,409 (22.9)	Middle	33,847 (60.2)	164,202 (58.4)	16,601 (57.2)	34,422 (54.3)
31,000–51,999	401 (16.1)	2,927 (19.6)	Top 20%	8,053 (14.3)	57,994 (20.6)	4,864 (16.7)	12,126 (19.1)
52,000–100,000	267 (10.7)	2,156 (14.5)	Unknown	102 (0.2)	7,251 (2.6)	69 (0.2)	7,435 (11.7)
>100,000	60 (2.4)	547 (3.7)					
Unknown	519 (20.9)	2,503 (16.8)					
Townsend deprivation index, mean (s.d.)	−0.8 (3.4)	−1.3 (3.1)					

**Fig. 1:**
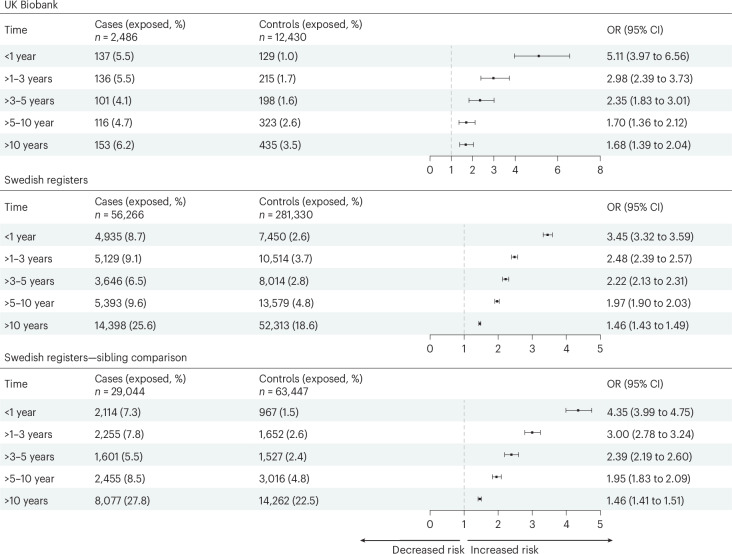
Association between previous hospital-treated infectious diseases and risk of epilepsy in older age. Estimates were derived from logistic regression conditioned on matching factors (age and sex), additionally adjusted for educational attainment, annual household income and Townsend deprivation index (UK Biobank only). Data points represent ORs; error bars represent 95% CIs. Source data

Cases of epilepsy exhibited a higher prevalence of infectious diseases across all time windows preceding the index date than controls (Fig. 1). In the UK Biobank, the odds ratio (OR) for infectious diseases during the year before the diagnosis of epilepsy was 5.11 (95% confidence interval (CI): 3.97–6.56). The OR during the 1–3 years and 3–5 years before diagnosis was 2.98 (95% CI: 2.39–3.73) and 2.35 (95% CI: 1.83–3.01), respectively. Notably, although the OR decreased to 1.68 (95% CI: 1.39–2.04) over 10 years before diagnosis, it remained statistically significant. A similar trend was observed in the Swedish registers, with an OR of 3.45 (95% CI: 3.32–3.59) for infectious diseases during the first year, 2.48 (95% CI: 2.39–2.57) during 1–3 years, 2.22 (95% CI: 2.13–2.31) during 3–5 years and 1.46 (95% CI: 1.43–1.49) during over 10 years before the diagnosis of epilepsy. Consistent findings were noted in the sibling analysis.

In the sensitivity analysis of the UK Biobank, similar results were obtained when restricting analysis to cases and controls with available primary care data (Extended Data Table 1). In the Swedish registers, similar results were noted for different subtypes of epilepsy (Extended Data Table 2). In the stratified analysis by history of brain tumor, we found 2,903 cases and 759 controls to have a history of brain tumor. A positive association was only noted among individuals without a history of brain tumor (OR: 1.95; 95% CI: 1.92–1.99) but not among those with such history (OR: 0.97; 95% CI: 0.82–1.15). The analysis combining clinical diagnosis and use of antiepileptic drugs to define epilepsy identified 46,382 cases, showing consistently a positive association between hospital-treated infectious diseases and epilepsy in all time windows before the diagnosis of epilepsy (Extended Data Table 3). The analyses identifying hospital-treated infectious diseases through outpatient care or prescribed use of anti-infectives also noted a positive association, although the magnitude of the association diminished slightly (Extended Data Table 4).

**Fig. 2:**
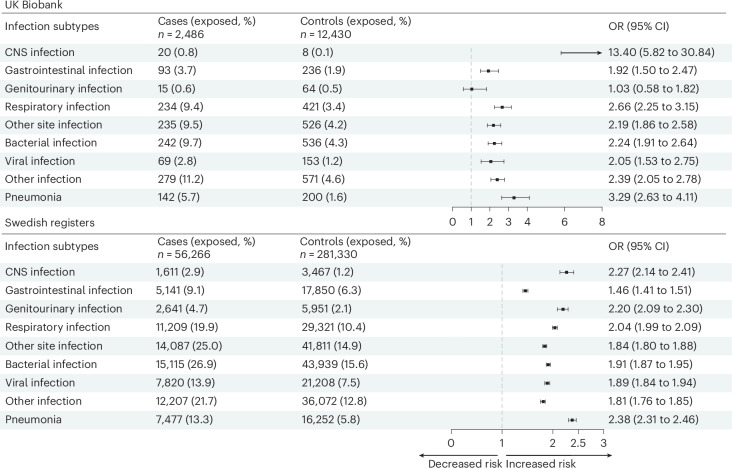
Association between previous hospital-treated infectious diseases and risk of epilepsy in older age by site and type of infection. Estimates were derived from logistic regression conditioned on matching factors (age and sex), additionally adjusted for educational attainment, annual household income and Townsend deprivation index (UK Biobank only). Data points represent ORs; error bars represent 95% CIs. Source data

In the UK Biobank, CNS infections exhibited the strongest association with epilepsy (OR: 13.40, 95% CI: 5.82–30.84; Fig. 2). A statistically significant association was also observed for non-CNS infections, with pneumonia showing the strongest association (OR: 3.29, 95% CI: 2.63–4.11). A positive association was noted for bacterial (OR: 2.24, 95% CI: 1.91–2.64), viral (OR: 2.05, 95% CI: 1.53–2.75) and other (OR: 2.39, 95% CI: 2.05–2.78) infections. These findings were corroborated in the Swedish registers where CNS infections (OR: 2.27, 95% CI: 2.14–2.41) and pneumonia (OR: 2.38, 95% CI: 2.31–2.46) demonstrated the strongest associations.

**Fig. 3:**
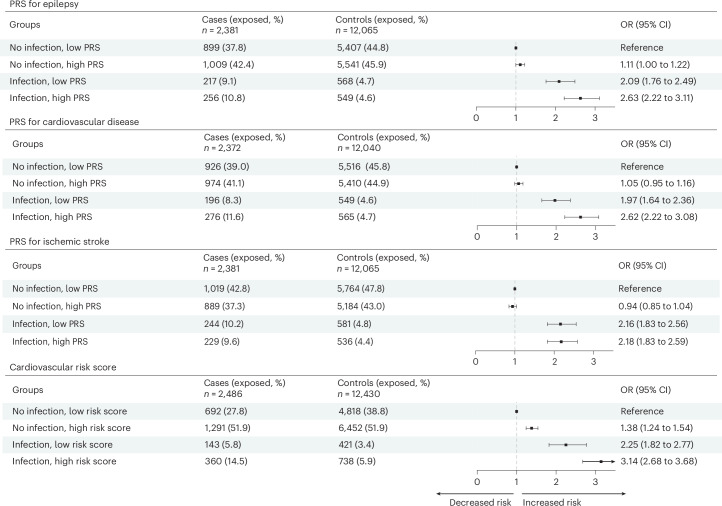
Hospital-treated infectious diseases, genetic susceptibility, cardiovascular risk score and risk of epilepsy in older age in the UK Biobank. Estimates were derived from logistic regression conditioned on matching factors (age and sex), additionally adjusted for educational attainment, annual household income and Townsend deprivation index. Data points represent ORs; error bars represent 95% CIs. Source data

In the UK Biobank, a high polygenic risk score (PRS) for epilepsy (OR: 1.11, 95% CI: 1.00–1.22) and a high cardiovascular risk score (OR: 1.38, 95% CI: 1.24–1.54) were associated with an elevated risk, whereas a high PRS for cardiovascular disease (CVD) or cerebrovascular disease was not associated with the risk of epilepsy in older age (Fig. 3). Individuals with infectious diseases and a high PRS for CVD had a greater risk increase (OR: 2.62, 95% CI: 2.22–3.08), compared to those with infectious diseases but a low PRS for CVD (OR: 1.97, 95% CI: 1.64–2.36), using individuals with no infectious disease and a low PRS as the reference (*P* for difference = 0.02). A similar result pattern was noted for PRS for epilepsy (OR: 2.63 versus OR: 2.09; *P* for difference = 0.06) and cardiovascular risk score (OR: 3.14 versus OR: 2.25; *P* for difference = 0.01), but not for the PRS of cerebrovascular disease. In the Swedish registers, individuals with infectious diseases and a history of CVD had a greater risk increase (OR: 4.77, 95% CI: 4.64–4.91), compared to those with infectious diseases but no history of CVD (OR: 1.86, 95% CI: 1.80–1.92), using individuals with neither infectious diseases nor CVD history as the reference (*P* for difference < 0.01; Extended Data Table 5).

This study represents a comprehensive analysis of the relationship between prior infectious diseases and the subsequent risk of epilepsy in older age. Leveraging data from two large study populations over the age of 50, we demonstrated a long-term impact of hospital-treated infectious diseases on the risk of late-onset epilepsy. Although the magnitude of the association diminished over time, the increased risk of epilepsy remained significant more than 10 years after infection. These findings were corroborated in a sibling analysis, suggesting the limited role of familial confounding, due to genetic, environmental and lifestyle factors shared between full siblings. Subgroup analyses revealed a consistently increased risk of epilepsy across various infection types and sites, suggesting a potentially generic role of severe infectious events in the development of late-onset epilepsy. Notably, we observed a greater risk increase among individuals with a previous infectious disease together with a high genetic susceptibility to epilepsy or CVD, a high cardiovascular risk score or a CVD history, compared to individuals with only a previous infectious disease, highlighting the potential joint effects between infectious diseases and cardiovascular vulnerabilities in the development of late-onset epilepsy.

There is currently no consensus on the definition of late-onset epilepsy^3^. Regardless, as the mean age of diagnosis was 66 years among cases with epilepsy in the UK Biobank and 70 years among cases in Swedish registers, our findings are mainly applicable to epilepsy in older age. In the analysis by site of infections, the strongest association was noted for CNS infections, corroborating that acute infectious diseases can cause epileptic seizures or acute symptomatic seizures^7–10^, whereas CNS infections may contribute to the long-term risk of epilepsy^14^. The positive associations noted for infections outside the CNS are in line with a previous study showing that infections not directly affecting the CNS might also modulate the risk of epilepsy in childhood^16^ and suggest that infectious diseases have a prolonged impact on the development of epilepsy in older age. It is also noteworthy that infections occurring at sites closer to the CNS (for example, pneumonia) showed a stronger association with epilepsy, compared to infections at more distant sites.

Interorgan communication offers a potential explanation for the long-lasting impact of infectious diseases on the risk of epilepsy in older age. CVD and stroke are common comorbidities among patients with epilepsy in older age; however, the nature of such comorbidity is not fully understood. Our study indicates that, while a genetic predisposition to CVD alone does not increase the risk of late-onset epilepsy, such genetic predisposition could potentially magnify the effect of other risk factors, for example, infectious diseases. Similarly, a high cardiovascular risk score, calculated based on an array of cardiovascular risk factors, as well as a previous diagnosis of CVD, also appeared to strengthen the association between infectious diseases and epilepsy. One explanation is that a high risk for CVD (due to genetic or modifiable risk factors) and a preexisting CVD may induce vascular or immune-response alterations that modulate the typical inflammatory responses to infections. Further, genetic susceptibility to CVD may amplify the vascular or inflammatory damage caused by infections, leading to a heightened risk of epilepsy. Alternatively, infection and its resultant inflammatory responses might exacerbate cardiovascular vulnerability, triggering the clinical onset of epilepsy. Regardless of the temporal order between events, infections, genetic predisposition and cardiovascular vulnerability might all contribute to the cause of epilepsy in older age.

An alternative explanation for the enduring effect of infectious diseases may involve the activation of macrophages and natural killer cells by an infectious event inside or outside the CNS, such as the lungs, leading to subsequent production of pro-inflammatory cytokines and nonspecific stimulation of pre-primed T cells^24,25^. Dysfunction of the blood–brain barrier (BBB) occurs in normal aging, resulting from the gradual loss of endothelial tight junctions^26^. Activated immune cells can traverse the BBB, especially in older age, instigating neuroinflammation and precipitating neurological disorders^24^. BBB changes have also been demonstrated in systemic inflammation and are related to various conditions like septic encephalopathy, neurodegenerative disorders, delirium and sickness behavior^27^. The finding that an association between hospital-treated infectious disease and epilepsy in older age was only noted among individuals without a previous history of brain tumor, benign or malignant, but not among individuals with such history might suggest that infections are more relevant for epilepsies without a clear cause. However, validation studies are needed to substantiate this finding.

A notable strength of the present study is the utilization of two independent study populations, namely the UK Biobank and the Swedish registers. Other strengths include the analysis of the temporal pattern of the association and the joint effects of infectious diseases with other risk factors for epilepsy, as well as the use of a sibling analysis in the Swedish registers to alleviate the concern of familial confounding. The study also has limitations. One concern in the UK Biobank analysis is the completeness of epilepsy ascertainment. While the positive predictive value (PPV) of a single read code diagnosis of epilepsy is high^28^, the sensitivity ranges from 83% to 88%^28^. This is likely attributed to different reasons, including the lack of outpatient care data and the fact that primary care data are available for only 45% of the participants. Further, as participation was completely voluntary and targeted at a specific age range, the study sample of the UK Biobank was younger and healthier than that of the Swedish registers. These limitations likely contributed to the different rates of hospital-treated infectious diseases and epilepsy noted between the two samples. As the analysis was always performed within each sample separately, such differences should not affect the interval validity of the sample-specific results; however, direct comparison of results between the study samples should be done with caution. There is also potential for surveillance bias. Patients with a hospital-treated infectious disease are more likely to be surveyed, particularly immediately following a hospitalization, and would therefore more likely receive a diagnosis of epilepsy than others. However, as we excluded cases with a hospital-treated infectious disease within 30 days before the epilepsy diagnosis, and that a persistently increased risk of epilepsy was noted more than 10 years after infection, it is unlikely that surveillance bias alone explains the results. Lastly, as our study defined infectious diseases through inpatient care (main analysis) or outpatient/primary care (sensitivity analysis), we identified relatively severe infectious diseases. It remains unclear whether mild infectious diseases not attended by healthcare are associated with an increased risk of late-onset epilepsy. The full spectrum of infectious diseases with different severity (spanning from asymptomatic to requiring intensive care use) and its impact on epilepsy thus remains to be investigated.

In conclusion, our results demonstrate a sustained impact of infectious diseases on risk of epilepsy in older age, irrespective of the type or location of infection. Our finding of a greater risk increment among individuals with both infectious diseases and a high genetic susceptibility to epilepsy or CVD, a high cardiovascular risk score or a CVD history suggests that eliminating cardiovascular risk factors may help lower the incidence of late-onset epilepsy.

## Methods

The analysis using data from the UK Biobank was authorized under UK Biobank application 76517 and approved by the Swedish Ethical Review Authority (DNR: 2022-01516-01). The analysis using data from the Swedish registers was approved by the Swedish Ethical Review Authority (DNR: 2012/1814-31/4).

### Data sources

#### UK Biobank

During 2006–2010, the UK Biobank recruited over 500,000 participants from 22 assessment centers in England, Scotland and Wales, collecting data on sociodemographic factors, lifestyle, medical history and electronic healthcare records through questionnaires, physical examinations and cross-linkages to national databases^29^. The UK Biobank received approval from the NHS Research Ethics Committee (REC reference: 16/NW/0274).

#### Swedish population and health registers

The Swedish Total Population Register includes demographic information for all residents of Sweden since the 1960s, including birth, death, migration, and so on. The Swedish Patient Register includes nationwide complete data on inpatient hospital care since 1987 and data on more than 80% of outpatient hospital visits since 2001. The Swedish Prescribed Drug Register includes nationwide data on prescribed medication use since July 2005 in Sweden^30^. The Swedish Multi-Generation Register includes largely complete information on familial links for all individuals born in Sweden since 1932. The Swedish Cancer Register includes nationwide complete information on all cancers newly diagnosed in Sweden since 1958. Individual-level data might be linked across different registers using the Swedish Personal Identity Number uniquely assigned to all residents in Sweden at the time of birth or immigration.

### Study design

We conducted a cohort study of the UK Biobank participants, starting follow-up from either the date of recruitment to the UK Biobank or their 50th birthday, whichever came later. We excluded participants who had withdrawn from the study or had a preexisting diagnosis of epilepsy identified through inpatient (category 2006; since 1997) or primary (available for approximately 45% of the participants; since 1938 in England, 1939 in Scotland, 1940 in England and 1948 in Wales) care records or through self-report at recruitment (field ID 20002: 1,264). The remaining participants were followed until a first diagnosis of epilepsy, death or end of follow-up (1 March 2020), whichever came first. We identified newly diagnosed cases of epilepsy using either a discharge diagnosis of epilepsy through an inpatient care episode (International Classification of Diseases, Tenth Revision (ICD-10) code G40) or a primary care diagnosis (read codes F25, 1O30, 667B and SC200). Although ICD codes in combination with antiepileptic drugs may demonstrate the greatest PPV in the ascertainment of epilepsy, we opted to use ICD codes together with read codes as data on medication use are not available for all participants of the UK Biobank and read codes have been shown to have a high PPV (93–98%)^28^.

We similarly conducted a cohort study using data from the Swedish Total Population Register, including all individuals who were born during 1932–1966 in Sweden whose parents were also born in Sweden. These individuals were followed from 1 January 2004 or their 50th birthday, whichever came later, until a first diagnosis of epilepsy, death or the end of follow-up (31 December 2016), whichever came first. We identified epilepsy through a hospital visit, inpatient or outpatient, concerning epilepsy (as the primary diagnosis or a secondary diagnosis), according to the Swedish Patient Register, using ICD-10 code G40, which was shown to have a PPV of around 90%^31^. Individuals who had a hospital visit concerning epilepsy between January 2001 (when data on outpatient hospital visits became available in the Patient Register) and the start of follow-up were excluded as we aimed to study newly diagnosed epilepsy. A washout period of 3 years was recommended in previous studies similarly using electronic healthcare data^32,33^. To assess the soundness of the results to the epilepsy definition, in a sensitivity analysis, we required the identified cases of epilepsy to also have prescribed use of antiepileptic drugs ascertained through Anatomical Therapeutic Chemical (ATC) code N03, according to the Swedish Prescribed Drug Register.

We then performed two nested case–control studies within the above cohorts to assess the impact of hospital-treated infectious diseases on the risk of epilepsy in older age. Newly diagnosed cases of epilepsy during the follow-up of both cohorts were included as cases. As we were primarily interested in the long-term impact of infectious diseases on risk of epilepsy, we excluded cases with a record of hospital-treated infectious diseases during the 30 days before diagnosis from the analysis, leaving 2,486 cases in the UK Biobank and 56,266 cases in the Swedish registers. For each case, we then randomly selected five age-matched and sex-matched controls from the cohorts, using the method of incidence density sampling to ensure that the case and their controls had the same exposure time window^34^. Date of diagnosis of the case was used as the index date for the case and their individually matched controls. In the nested case–control study using the Swedish registers, we additionally conducted a sibling analysis to assess the potential influence of familial confounding, due to genetic or nongenetic factors shared between full siblings, on the studied association. For this purpose, we identified full siblings of the cases through the Swedish Multi-Generation Register. This sibling analysis included 29,044 cases and their 63,447 full siblings who were free of epilepsy on the index date (that is, unaffected full siblings).

### Infectious diseases

We identified all hospital-treated infectious diseases before the index date of the cases and their individually matched controls (or unaffected siblings) through the hospital inpatient data in the UK Biobank and the Swedish Patient Register as the exposure of interest. We used inpatient care episodes where an infectious disease was indicated as the primary discharge diagnosis to ascertain such exposure. We first studied any hospital-treated infectious disease, and then categorized infectious diseases by site (CNS, genitourinary, respiratory or other/unspecified) and type (bacterial, viral or other). We studied pneumonia as a separate entity. The specific ICD codes used to identify infectious diseases^35^ are shown in Supplementary Table 1.

To assess the soundness of study results to the definition of inpatient-treated infectious disease, in a sensitivity analysis of the Swedish registers, we also defined hospital-treated infectious diseases through specialized outpatient care (via the Swedish Patient Register) and the use of anti-infective medications (ATC codes J01–J05 and P01–P03; via the Swedish Prescribed Drug Register) as proxies for milder infections. In contrast to the Patient Register, the Prescribed Drug Register includes information on medications prescribed by both specialized and primary care in Sweden.

### PRSs

The genotyping methodologies and arrays used in the UK Biobank have been elaborated previously^36^. In this study, we used PRSs for epilepsy, CVD and ischemic stroke as proxies for genetic susceptibility to epilepsy, CVD and cerebrovascular disease, respectively. A PRS for CVD was provided by the UK Biobank (field ID 26223), whereas PRSs for epilepsy and ischemic stroke were computed with single nucleotide polymorphisms identified in previous genome-wide association studies^37,38^, using a weighted approach as detailed earlier^39^. Cases and controls of the nested case–control study in the UK Biobank were then classified as having a high or low PRS of epilepsy, CVD or cerebrovascular disease according to the median PRSs of these phenotypes in the entire UK Biobank^40^. PLINK 2 (www.cog-genomics.org/plink/2.0/) was used in these analyses^40^.

### Cardiovascular risk score and history of CVD

In the UK Biobank, we assessed the cardiovascular risk score of the cases and controls through a specified scoring system using information collected at recruitment^41^, where points were allocated based on hypertension treatment (1 point for yes), elevated blood pressure (1 point for yes), cholesterol medication (1 point for yes), diabetes diagnosis (1 point for yes), waist-to-hip ratio surpassing gender-specific thresholds (1 point for yes) and smoking history (1 point for 10–50 pack-years, and 2 points for over 50 pack-years). The *APOE* e4 allele status also contributed to the score (1 point for a single e4 allele, and 2 points for double e4 alleles). Cases and controls were categorized as with a low (0–1 point) or high (2 or more points) cardiovascular risk score, based on the median value of the entire UK Biobank. In the Swedish registers, we identified a diagnosis of CVD before the index date, via a hospital visit concerning CVD according to the Swedish Patient Register, for the cases and controls. ICD-8 codes 390–458, ICD-9 codes 390–459 and ICD-10 codes I00–I99 were used to ascertain CVD history before the index date.

### Statistical analysis

We used logistic regression conditioned on matching factors (age and sex), yielding ORs with 95% CIs to estimate the association between previous hospital-treated infectious diseases and risk of epilepsy over age 50. As covariates, we included educational attainment (UK Biobank: college degree, A-level, O-level, Certificate of Secondary Education or equivalent, National Vocation Qualifications or equivalent, other professional qualifications, or unknown; Swedish registers: >12 years, 9–12 years, <9 years, or unknown), annual household income (UK Biobank: <£18,000, £18,000–30,999, £31,000–51,999, £52,000–100,000, >£100,000 or unknown; Swedish registers: lowest 20%, middle, top 20% or unknown) and Townsend deprivation index (as a continuous variable, UK Biobank only). Age and sex were additionally adjusted in the sibling analysis of the Swedish registers as cases and their unaffected full siblings were matched by family identifiers. We studied hospital-treated infectious diseases experienced in different time windows before the index date (>3 months to 1 year, >1–3 years, >3–5 years, >5–10 years or >10 years).

In the UK Biobank, to assess the potential influence of having primary care data for only a proportion of the study participants, we performed a sensitivity analysis restricted to participants with available primary care data. In the Swedish registers, we conducted several sensitivity analyses in relation to epilepsy. First, to evaluate whether the observed association would vary across clinical subtypes, we performed separate analyses for focal epilepsy (ICD-10: G40.0, G40.1, G40.2), generalized epilepsy (ICD-10: G40.3, G40.4), special epileptic syndromes (ICD-10: G40.5), unspecified generalized seizures (ICD-10: G40.6, G40.7) and other types (ICD-10: G40.8, G40.9). Second, to examine whether the association would vary by potential etiologies of epilepsy, we conducted separate analyses for epilepsy with a previous history of brain tumor (via the Swedish Cancer Register, ICD-7: 193). Finally, we restricted the analysis to cases with both an epilepsy diagnosis and a record of prescribed use of antiepileptic drugs. Given the availability of data in the Prescribed Drug Register, the last sensitivity analysis was restricted to cases and their controls whose index date was during July 2005–December 2016.

In addition to any infectious disease, we also examined different infectious diseases (that is, by site and type of infection as well as for pneumonia). In the UK Biobank, we assessed potential joint effects between infectious diseases and genetic susceptibility to epilepsy or cardiovascular and cerebrovascular diseases, through categorizing the study participants into four groups: (1) with no infectious disease and low PRS (reference group); (2) with no infectious disease but a high PRS; (3) with infectious disease and a low PRS; and (4) with infectious disease and a high PRS. We assessed the joint effects of infectious disease and cardiovascular risk score measured at recruitment to the UK Biobank in a similar manner. In the Swedish registers, we evaluated the joint effects of hospital-treated infectious diseases and history of CVD through categorizing the cases and controls into four groups where the group with neither infectious disease nor history of CVD was used as the reference. The difference between subgroup ORs was assessed by the Wald test^42^. The statistical significance of the interactions between hospital-treated infectious diseases and PRS, cardiovascular risk score and history of CVD was assessed in independent interaction models.

All analyses were conducted by Python (version 3.10), R (version 4.3) and SAS (version 9.4) software. A two-sided *P* < 0.05 was considered statistically significant.

### Reporting summary

Further information on research design is available in the Nature Portfolio Reporting Summary linked to this article.

## Supplementary information

Supplementary Information Supplementary Table 1.

Reporting Summary

## Source data

Source Data Fig. 1 Statistical source data used to generate the forest plots and regression results in Fig. 1. This includes ORs and 95% CIs from conditional logistic regression analyses.

Source Data Fig. 2 Statistical source data used to generate the forest plots and regression results in Fig. 2. This includes ORs and 95% CIs from conditional logistic regression analyses.

Source Data Fig. 3 Statistical source data used to generate the forest plots and regression results in Fig. 3. This includes ORs and 95% CIs from conditional logistic regression analyses.

## Figures and Tables

**Extended Data Table 1:**
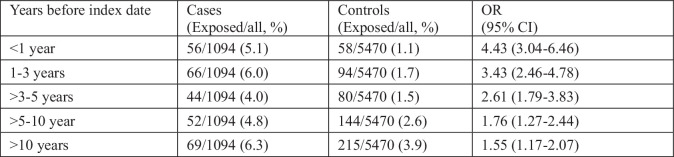
Association between previous hospital-treated infectious diseases and risk of epilepsy - sensitivity analysis of the UK Biobank restricted to individuals with available primary care data

Analysis was restricted to individuals in the UK Biobank with available primary care data. Estimates were derived from logistic regression conditioned on matching factors (age and sex), additionally adjusted for educational attainment, annual household income, and Townsend deprivation index. Data points represent ORs; error bars represent 95% CIs. CI, confidence interval; OR, odds ratio.

**Extended Data Table 2:**
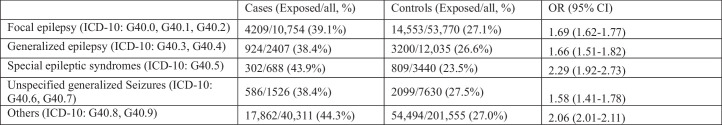
Association between previous hospital-treated infectious disease and the risk of epilepsy – analysis by subtypes of epilepsy in the Swedish registers

Estimates were derived from logistic regression conditioned on matching factors (age and sex), additionally adjusted for educational attainment, annual household income. Data points represent ORs; error bars represent 95% CIs. CI, confidence interval; OR, odds ratio.

**Extended Data Table 3:**
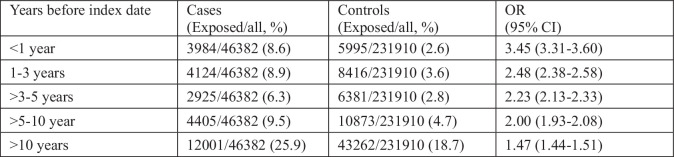
Association between previous infections and risk of epilepsy - sensitivity analysis of the Swedish registers restricted to cases with prescribed use of antiepileptic drugs

Estimates were derived from logistic regression conditioned on matching factors (age and sex), additionally adjusted for educational attainment, annual household income. Data points represent ORs; error bars represent 95% CIs. CI, confidence interval; OR, odds ratio.

**Extended Data Table 4:**
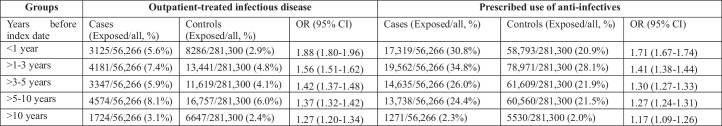
Association between previous outpatient-treated infectious disease or prescribed use of anti-infectives and risk of epilepsy - sensitivity analysis of the Swedish registers

Estimates were derived from logistic regression conditioned on matching factors (age and sex), additionally adjusted for educational attainment, annual household income. Data points represent ORs; error bars represent 95% CIs. CI, confidence interval; OR, odds ratio.

**Extended Data Table 5:**
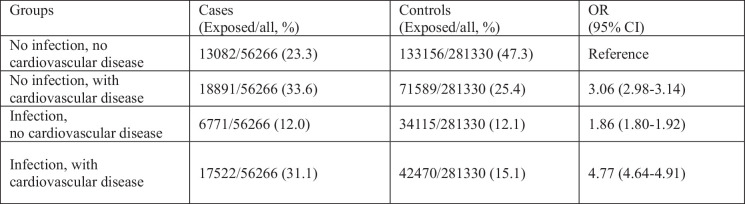
Infections, cardiovascular disease, and risk of epilepsy – analysis of the Swedish registers

Estimates were derived from logistic regression conditioned on matching factors (age and sex), additionally adjusted for educational attainment, annual household income. Data points represent ORs; error bars represent 95% CIs. CI, confidence interval; OR, odds ratio.

## Data Availability

Data from the UK Biobank (http://www.ukbiobank.ac.uk/) are available to all researchers upon making an application. Swedish registers retain individual-level data, which are not openly accessible in accordance with Swedish and European legislations. Nevertheless, researchers can gain access to the data through securing ethical clearance and via contact with the registers (https://www.socialstyrelsen.se/en/statistics-and-data/registers/).

## Code availability

No custom code was developed specifically for this study. The analysis and figure-generation scripts used to produce the results reported in this study have been deposited in Code Ocean.

## References

[1] Fisher, R. S. et al. ILAE official report: a practical clinical definition of epilepsy. *Epilepsia* **55**, 475–482 (2014). 24730690 10.1111/epi.12550

[2] Goldberg, E. M. & Coulter, D. A. Mechanisms of epileptogenesis: a convergence on neural circuit dysfunction. *Nat. Rev. Neurosci.* **14**, 337–349 (2013). 23595016 10.1038/nrn3482 PMC3982383

[3] Sen, A., Jette, N., Husain, M. & Sander, J. W. Epilepsy in older people. *Lancet* **395**, 735–748 (2020). 32113502 10.1016/S0140-6736(19)33064-8

[4] Fiest, K. M. et al. Prevalence and incidence of epilepsy: a systematic review and meta-analysis of international studies. *Neurology* **88**, 296–303 (2017). 27986877 10.1212/WNL.0000000000003509 PMC5272794

[5] Beghi, E. & Giussani, G. Aging and the epidemiology of epilepsy. *Neuroepidemiology* **51**, 216–223 (2018). 30253417 10.1159/000493484

[6] Beghi, E. et al. The epidemiology of epilepsy in older adults: a narrative review by the ILAE Task Force on Epilepsy in the Elderly. *Epilepsia* **64**, 586–601 (2023). 36625133 10.1111/epi.17494

[7] Kotsopoulos, I. A., van Merode, T., Kessels, F. G., de Krom, M. C. & Knottnerus, J. A. Systematic review and meta-analysis of incidence studies of epilepsy and unprovoked seizures. *Epilepsia* **43**, 1402–1409 (2002). 12423392 10.1046/j.1528-1157.2002.t01-1-26901.x

[8] McKelvie, S., Moore, A., Croxson, C., Lasserson, D. S. & Hayward, G. N. Challenges and strategies for general practitioners diagnosing serious infections in older adults: a UK qualitative interview study. *BMC Fam. Pract.* **20**, 56 (2019). 31027482 10.1186/s12875-019-0941-8 PMC6486693

[9] Vezzani, A. et al. Infections, inflammation and epilepsy. *Acta Neuropathol.* **131**, 211–234 (2016). 26423537 10.1007/s00401-015-1481-5 PMC4867498

[10] Ramantani, G. & Holthausen, H. Epilepsy after cerebral infection: review of the literature and the potential for surgery. *Epileptic Disord.* **19**, 117–136 (2017). 28637636 10.1684/epd.2017.0916

[11] Singhi, P. Infectious causes of seizures and epilepsy in the developing world. *Dev. Med. Child Neurol.* **53**, 600–609 (2011). 21518343 10.1111/j.1469-8749.2011.03928.x

[12] Sander, J. W. & Perucca, E. Epilepsy and comorbidity: infections and antimicrobials usage in relation to epilepsy management. *Acta Neurol. Scand. Suppl.* **180**, 16–22 (2003). 14510816 10.1034/j.1600-0404.108.s180.3.x

[13] Zelano, J. & Westman, G. Epilepsy after brain infection in adults: a register-based population-wide study. *Neurology* **95**, e3213–e3220 (2020). 32989110 10.1212/WNL.0000000000010954

[14] Bodilsen, J., Dalager-Pedersen, M., van de Beek, D., Brouwer, M. C. & Nielsen, H. Long-term mortality and epilepsy in patients after brain abscess: a nationwide population-based matched cohort study. *Clin. Infect. Dis.* **71**, 2825–2832 (2020). 31773138 10.1093/cid/ciz1153

[15] Tran, V. T. A., Lee, L. P. & Cho, H. Neuroinflammation in neurodegeneration via microbial infections. *Front. Immunol.* **13**, 907804 (2022). 36052093 10.3389/fimmu.2022.907804 PMC9425114

[16] Olsen, M. et al. Hospital-diagnosed pertussis infection in children and long-term risk of epilepsy. *JAMA* **314**, 1844–1849 (2015). 26529162 10.1001/jama.2015.13971

[17] International League Against Epilepsy Consortium on Complex Epilepsies. Genome-wide mega-analysis identifies 16 loci and highlights diverse biological mechanisms in the common epilepsies. *Nat. Commun.* **9**, 5269 (2018). 30531953 10.1038/s41467-018-07524-z PMC6288131

[18] Wang, J. et al. Epilepsy and long-term risk of arrhythmias. *Eur. Heart J.* **44**, 3374–3382 (2023). 37602368 10.1093/eurheartj/ehad523 PMC10499547

[19] Olafsson, E. et al. Incidence of unprovoked seizures and epilepsy in Iceland and assessment of the epilepsy syndrome classification: a prospective study. *Lancet Neurol.* **4**, 627–634 (2005). 16168931 10.1016/S1474-4422(05)70172-1

[20] Husein, N., Josephson, C. B. & Keezer, M. R. Understanding cardiovascular disease in older adults with epilepsy. *Epilepsia* **62**, 2060–2071 (2021). 34245013 10.1111/epi.16991

[21] Tai, X. Y. et al. Association of dementia risk with focal epilepsy and modifiable cardiovascular risk factors. *JAMA Neurol.* **80**, 445–454 (2023). 36972059 10.1001/jamaneurol.2023.0339 PMC10043806

[22] Sun, M. et al. Piezo1-mediated neurogenic inflammatory cascade exacerbates ventricular remodeling after myocardial infarction. *Circulation* **149**, 1516–1533 (2024). 10.1161/CIRCULATIONAHA.123.065390 38235590

[23] Carnevale, D. Neuroimmune axis of cardiovascular control: mechanisms and therapeutic implications. *Nat. Rev. Cardiol.* **19**, 379–394 (2022). 35301456 10.1038/s41569-022-00678-w

[24] Odoardi, F. et al. T cells become licensed in the lung to enter the central nervous system. *Nature* **488**, 675–679 (2012). 22914092 10.1038/nature11337

[25] Chen, S. et al. Macrophages in immunoregulation and therapeutics. *Signal Transduct. Target. Ther.* **8**, 207 (2023). 37211559 10.1038/s41392-023-01452-1 PMC10200802

[26] Elahy, M. et al. Blood-brain barrier dysfunction developed during normal aging is associated with inflammation and loss of tight junctions but not with leukocyte recruitment. *Immun. Ageing* **12**, 2 (2015). 25784952 10.1186/s12979-015-0029-9 PMC4362825

[27] Varatharaj, A. & Galea, I. The blood-brain barrier in systemic inflammation. *Brain Behav. Immun.* **60**, 1–12 (2017). 26995317 10.1016/j.bbi.2016.03.010

[28] Mbizvo, G. K. et al. The accuracy of using administrative healthcare data to identify epilepsy cases: a systematic review of validation studies. *Epilepsia* **61**, 1319–1335 (2020). 32474909 10.1111/epi.16547

[29] Sudlow, C. et al. UK biobank: an open access resource for identifying the causes of a wide range of complex diseases of middle and old age. *PLoS Med.* **12**, e1001779 (2015). 25826379 10.1371/journal.pmed.1001779 PMC4380465

[30] Ludvigsson, J. F. et al. Registers of the Swedish total population and their use in medical research. *Eur. J. Epidemiol.* **31**, 125–136 (2016). 26769609 10.1007/s10654-016-0117-y

[31] Sveinsson, O., Andersson, T., Carlsson, S. & Tomson, T. The incidence of SUDEP: a nationwide population-based cohort study. *Neurology* **89**, 170–177 (2017). 28592455 10.1212/WNL.0000000000004094

[32] Lowerison, M. W. et al. Association of levels of specialized care with risk of premature mortality in patients with epilepsy. *JAMA Neurol.* **76**, 1352–1358 (2019). 31380987 10.1001/jamaneurol.2019.2268 PMC6686748

[33] Bakaki, P. M., Koroukian, S. M., Jackson, L. W., Albert, J. M. & Kaiboriboon, K. Defining incident cases of epilepsy in administrative data. *Epilepsy Res.* **106**, 273–279 (2013). 23791310 10.1016/j.eplepsyres.2013.05.005 PMC3759552

[34] Pearce, N. Incidence density matching with a simple SAS computer program. *Int. J. Epidemiol.* **18**, 981–984 (1989). 2621036 10.1093/ije/18.4.981

[35] Xu, Y. et al. Hospital-diagnosed infections before age 20 and risk of a subsequent multiple sclerosis diagnosis. *Brain* **144**, 2390–2400 (2021). 33693538 10.1093/brain/awab100

[36] Bycroft, C. et al. The UK Biobank resource with deep phenotyping and genomic data. *Nature* **562**, 203–209 (2018). 30305743 10.1038/s41586-018-0579-z PMC6786975

[37] O’Sullivan, J. W. et al. Combining clinical and polygenic risk improves stroke prediction among individuals with atrial fibrillation. *Circ. Genom. Precis. Med.* **14**, e003168 (2021). 34029116 10.1161/CIRCGEN.120.003168 PMC8212575

[38] Mars, N. et al. Systematic comparison of family history and polygenic risk across 24 common diseases. *Am. J. Hum. Genet.* **109**, 2152–2162 (2022). 36347255 10.1016/j.ajhg.2022.10.009 PMC9748261

[39] Rutten-Jacobs, L. C. et al. Genetic risk, incident stroke, and the benefits of adhering to a healthy lifestyle: cohort study of 306,473 UK Biobank participants. *BMJ* **363**, k4168 (2018). 30355576 10.1136/bmj.k4168 PMC6199557

[40] Chang, C. C. et al. Second-generation PLINK: rising to the challenge of larger and richer datasets. *Gigascience* **4**, 7 (2015). 25722852 10.1186/s13742-015-0047-8 PMC4342193

[41] Veldsman, M. et al. Cerebrovascular risk factors impact frontoparietal network integrity and executive function in healthy ageing. *Nat. Commun.* **11**, 4340 (2020). 32895386 10.1038/s41467-020-18201-5 PMC7477206

[42] Altman, D. G. & Bland, J. M. Interaction revisited: the difference between two estimates. *BMJ* **326**, 219 (2003). 12543843 10.1136/bmj.326.7382.219 PMC1125071

