# Supplementary material for: Hospital-treated infectious diseases and the risk of epilepsy in older age
Source: Nat Aging. 2025 Nov 4;5(11):2188–96. doi: 10.1038/s43587-025-01005-x (PMC12618234; doi:10.1038/s43587-025-01005-x)
Supplement: Supplementary file 1 — Supplementary Table 1. [file 43587_2025_1005_MOESM1_ESM.pdf]

nature aging

Supplementary information https://doi.org/10.1038/s43587-025-01005-x

Hospital-treated infectious diseases and the risk of epilepsy in older age

In the format provided by the authors and unedited

Supplementary Table 1. International Classification of Disease (ICD) codes, eighth (ICD-8), ninth (ICD-9) and tenth (ICD-10) revisions, for ascertainment of infections

**Infection**	**ICD revision**	**ICD codes**
Site of infection		
CNS	8	013,062,063,064,065,066,071,094,292,320,323,324, 390,474,040-046
	9	013,062,063,064,071,094,320,323,326,392,045-049
	10	A17,A80-A89,G00,G01,G02,G04,G05,I02
Gastrointestinal	8	014,123,127,129,540,567,000-009
	9	014,123,127,129,540,567,001-009
	10	A00-A09,B71,B81,B82,K35,K65,K67
Genitourinary	8	016,590,595,597
	9	016,590,595,597
	10	N30,N34,O23
Respiratory	8	010,011,012,033,034,075,115,116,490,501,503,510,460-466,470-474,480-486
	9	010,011,012,033,034,075,115,116,473,475,487,490,510, 460-466,480-486
	10	A15,A16,A37,A38,B27,B39,B40,B41,B42,B44,B59,J00-J06,J10,J12-J18,J20-J22,J32,J36,J40-J42,J86,P23
Other/unspecified	8	015,017,018,019,020-027,030,031,032,035,036,037,038,039, 050-057,060,061,067,068,070, 072, 073,074,076,077,078,079,080-089,090-093,095-099,100-104,110,111,112,113,114,117,121,122,124-126,128,129,130-136, 360, 362,363,380,381,382,383,391,392,420,421,422, 610-616,620,622,630,635,670,678,680-686,710,720,732,763,

		Y41
	9	015,017,018,020-027,030,031,032,035,036,037,038,039,040,041,050-057,060,061,065,066,070, 072,073,074,076,077,078,079,080-088,090-093,095-099,100-104,110,111,112,113,114,117,118,120,121,122,124-126, 128,130-136,137-139, 370,372,381,382,383,390,391,420,421,422, 614-616,647,670,675,680-686,711,730,771
	10	A18,A19,A20-A28,A30-A36,A39-A49,A50-A60,A63,A64,A65-A69,A70-A74,A75-A79,A90-A99, B00-B09,B15-B19,B20-B24,B25,B26,B28-B34,B35,B36,B37,B38,B43,B45,B46,B47,B48,B49,B50-B58,B60-B64,B65-B70,B72-B80,B83,B85-B89,B90-B94,B95,B96,B97,B99,G03,H10,H16,H32,H60,H70,I00, I01,I30,I33,I40,L00-L08,M00,M01,M02,M03,M86,N70-N77,O85,O86,O91,O98,P35-P39,V02,Z21,Z22
Type of infection		
Bacterial	8	595,597,612,613,614,616,620,622,630,635,670,678,680,000,001,002,003,004,005,073,076,080,081,082,083,320,362,380,381,382,383,421,461,481,482,501,510,567,590,681,682,684,710,720,010-019,020-027,030-039,090-099,100-104,390-392
	9	001,002,003,004,005,073,076,077,078,079,080,081,082,083,320,381,382,383,383,421,461,475,481,482,510,567,590,595,597,670,730,010-018,020-027,030-041,090-099,100-104,390-392,614-616,680-686
	10	A00,A01,A02,A03,A04,A05,A15-A19,A20-A28,A30-A49,A50-A58,A65-A69,A70-A74,A75-A79,B95,B96, G00,G01,H60,H70,I00-I02,I33,J01,J13,J14,J15,J36,J86,K65,L00-L08,M00,M86,N30,N34,N70-N77,O23,O85,O86,P36
Viral	8	075,360,420,422,460,464,465,466,480,040-046,050-057,060-068,070-079,470-474
	9	070,071,072,074,075,077,078,079,372,420,422,460,464,465,466,480,487,647,711,045-049,050-057,060-066
	10	A08,A60,A80-A89,A90-A99,B00-B09,B15-B19,B20-B24,B25-B34,B27,B97,B99,H10,I30,I40,J00,J04,

		J05,J06,J10,J12,J20,J21,O98,P35,Z21
Other	8	006,007,008,009,084,085,086,087,088,089,110-117,120-129,130-136,292,323,324,363,462,463,483,484,485,486,490,503,540,610,611,615,683,685,686,732,763,Y41
	9	006,007,008,009,084,085,086,087,088,110-118,120-129, 130-136,137-139,323,326,370,462,463,473,483,484,485,486,490,540,675,771
	10	A06,A07,A09,A59,A63,A64,B35-B49,B50-B64,B65-B83,B85-B89,B90-B94,G02,G04,G05,H16,H32,J02,J03,J16,J17,J18,J22,J32,J40,J41,J42,K35,K67,M01,M02,M03,O91,P23,P37,P38,P39,V02,Z22
Specific infection		
Pneumonia	8	480-486
	9	480-486
	10	J12-J18, P23
