# Supplementary material for: Hospital-treated infectious diseases and the risk of epilepsy in older age
Source: Nat Aging. 2025 Nov 4;5(11):2188–96. doi: 10.1038/s43587-025-01005-x (PMC12618234; doi:10.1038/s43587-025-01005-x)
Supplement: Supplementary file 2 — Reporting Summary [file 43587_2025_1005_MOESM2_ESM.pdf]

nature portfolio

Corresponding author(s): Fang Fang

Last updated by author(s): Mar 6, 2025

Reporting Summary

Nature Portfolio wishes to improve the reproducibility of the work that we publish. This form provides structure for consistency and transparency in reporting. For further information on Nature Portfolio policies, see our Editorial Policies and the Editorial Policy Checklist.

Statistics

For all statistical analyses, confirm that the following items are present in the figure legend, table legend, main text, or Methods section.

n/a	Confirmed	
☐	☒	The exact sample size (*n*) for each experimental group/condition, given as a discrete number and unit of measurement
☐	☒	A statement on whether measurements were taken from distinct samples or whether the same sample was measured repeatedly
☐	☒	The statistical test(s) used AND whether they are one- or two-sided<br>*Only common tests should be described solely by name; describe more complex techniques in the Methods section.*
☐	☒	A description of all covariates tested
☐	☒	A description of any assumptions or corrections, such as tests of normality and adjustment for multiple comparisons
☐	☒	A full description of the statistical parameters including central tendency (e.g. means) or other basic estimates (e.g. regression coefficient) AND variation (e.g. standard deviation) or associated estimates of uncertainty (e.g. confidence intervals)
☐	☒	For null hypothesis testing, the test statistic (e.g. F, t, r) with confidence intervals, effect sizes, degrees of freedom and P value noted<br>*Give P values as exact values whenever suitable.*
☒	☐	For Bayesian analysis, information on the choice of priors and Markov chain Monte Carlo settings
☒	☐	For hierarchical and complex designs, identification of the appropriate level for tests and full reporting of outcomes
☐	☒	Estimates of effect sizes (e.g. Cohen's *d*, Pearson's *r*), indicating how they were calculated

Our web collection on statistics for biologists contains articles on many of the points above.

Software and code

Policy information about availability of computer code

Data collection	All analyses were conducted by Python software (version 3.10), R software (version 4.3), and SAS 9.4. Custom code were used.
Data analysis	All analyses were conducted by Python software (version 3.10), R software (version 4.3), and SAS 9.4. Custom code were used.

For manuscripts utilizing custom algorithms or software that are central to the research but not yet described in published literature, software must be made available to editors and reviewers. We strongly encourage code deposition in a community repository (e.g. GitHub). See the Nature Portfolio guidelines for submitting code & software for further information.

Data

Policy information about availability of data

All manuscripts must include a data availability statement. This statement should provide the following information, where applicable:

- Accession codes, unique identifiers, or web links for publicly available datasets
- A description of any restrictions on data availability
- For clinical datasets or third party data, please ensure that the statement adheres to our policy

Data from the UK Biobank (http://www.ukbiobank.ac.uk/) are available to all researchers upon making an application. Swedish registers retain the original data, which is not openly accessible in accordance with Swedish and European legislations. Nevertheless, researchers can gain access to the data through securing ethical clearance and via contact with the registries.

Research involving human participants, their data, or biological material

Policy information about studies with human participants or human data. See also policy information about sex, gender (identity/presentation), and sexual orientation and race, ethnicity and racism.

Reporting on sex and gender	Sex (biological attribute) was derived from the UK Biobank and the Swedish population register and included as a covariate in our analyses to adjust for potential confounding. No separate data on gender identity was collected or analyzed. Due to GDPR and the respective data providers' policies, individual-level data cannot be shared publicly.
Reporting on race, ethnicity, or other socially relevant groupings	Not applicable
Population characteristics	We analyzed individuals aged 50 years or older from the UK Biobank and the Swedish national registers. In the UK Biobank, 2,486 participants with newly diagnosed epilepsy were matched by age and sex to 12,430 controls. In the Swedish registers, 56,266 cases were matched to 281,330 population controls, and 29,044 cases were matched to 63,447 sibling controls. Detailed population characteristics including age, sex, educational attainment, annual household income and Townsend deprivation index could be found in Table 1.
Recruitment	Participants were identified from the UK Biobank and Swedish registers. In the UK Biobank, individuals voluntarily enrolled between 2006 and 2010, which may introduce self-selection bias, as participants tend to be healthier and more health-conscious than the general population. In the Swedish registers, cases of newly diagnsoed epilepsy were identified using national healthcare records, with population and sibling controls selected through register-based matching. As register-based data collection is comprehensive, selection bias is minimized in the Swedish cohort.
Ethics oversight	UK Biobank received approval from the NHS Research Ethics Committee (REC reference: 16/NW/0274). The analysis using data from the UK Biobank was authorized under UK Biobank application 76517 and approved by the Swedish Ethical Review Authority (DNR: 2022-01516-01). The analysis using data from the Swedish registers was approved by the Swedish Ethical Review Authority (DNR: 2012/1814-31/4).

Note that full information on the approval of the study protocol must also be provided in the manuscript.

Field-specific reporting

Please select the one below that is the best fit for your research. If you are not sure, read the appropriate sections before making your selection.

☒ Life sciences ☐ Behavioural & social sciences ☐ Ecological, evolutionary & environmental sciences

For a reference copy of the document with all sections, see nature.com/documents/nr-reporting-summary-flat.pdf

Life sciences study design

All studies must disclose on these points even when the disclosure is negative.

Sample size	The sample size was determined by the availability of eligible individuals in the UK Biobank and Swedish national registers. In the UK Biobank, 2,486 cases of incident epilepsy were matched with 12,430 age- and sex-matched controls, while in the Swedish registers, 56,266 cases were matched with 281,330 population controls, and 29,044 cases were matched with 63,447 sibling controls. No formal sample size calculation was performed, as the study utilized all available data from these large-scale population-based cohorts.
Data exclusions	In the UK Biobank analysis, we excluded participants who had withdrawn from the study or had a pre-existing diagnosis of epilepsy identified through inpatient (Category 2006; since year 1997) or primary (available for approximately 45% of the UK Biobank participants; since 1938 in England, 1939 in Scotland, 1940 in England, and 1948 in Wales) care records or through self-report at start of follow-up (Field ID 20002: 1264). In the Swedish register analysis, individuals who had a hospital visit concerning epilepsy between January 2001 (when data on outpatient hospital visits became available in the Patient Register) and start of follow-up were excluded as we aimed to study incident epilepsy.
Replication	The findings were replicated across two independent datasets: the UK Biobank and the Swedish national registers, both showing a consistent association between hospital-treated infectious diseases and the risk of epilepsy over age 50. Further validation was conducted through sibling comparisons, which helped account for shared familial and genetic factors. The consistency of results across these different study designs supports the robustness and reproducibility of our findings.
Randomization	A nested case-control study design was applied, with covariates such as educational attainment, annual household income, and the Townsend Deprivation Index (UK Biobank only) adjusted in the statistical model. Age and sex were accounted for in the conditional logistic regression.
Blinding	Data was collected prospectively, while the current study is a retrospective analysis; therefore, blinding is not applicable.

Reporting for specific materials, systems and methods

We require information from authors about some types of materials, experimental systems and methods used in many studies. Here, indicate whether each material, system or method listed is relevant to your study. If you are not sure if a list item applies to your research, read the appropriate section before selecting a response.

Materials & experimental systems

n/a	Involved in the study
☒	☐ Antibodies
☒	☐ Eukaryotic cell lines
☒	☐ Palaeontology and archaeology
☒	☐ Animals and other organisms
☐	☒ Clinical data
☒	☐ Dual use research of concern
☒	☐ Plants

Methods

n/a	Involved in the study
☒	☐ ChIP-seq
☒	☐ Flow cytometry
☒	☐ MRI-based neuroimaging

Clinical data

Policy information about clinical studies

All manuscripts should comply with the ICMJE guidelines for publication of clinical research and a completed CONSORT checklist must be included with all submissions.

Clinical trial registration

Observational study; Not applicable

Study protocol

Observational study; Not applicable

Data collection

Data were obtained from the UK Biobank and the Swedish national registers. The UK Biobank recruited participants between 2006 and 2010, collecting extensive health, lifestyle, genetic, and medical record data from individuals aged 40-69 years at baseline. The Swedish national registers provide prospectively collected nationwide healthcare data, including hospital admissions, diagnoses, and history of prescriptions, with epilepsy cases identified through the National Patient Register. Data linkage to registries enabled long-term follow-up of participants.

Outcomes

In UK Biobank analysis, we identified newly diagnosed cases of epilepsy using either a discharge diagnosis of epilepsy through an inpatient care episode (International Classification of Diseases [ICD] -10 code G40) or primary care diagnosis (Read codes F25, 1O30, 667B, and SC200). In Swedish register analysis, we identified epilepsy through a hospital visit, inpatient or outpatient, concerning epilepsy (as the primary diagnosis or a secondary diagnosis), according to the Swedish Patient Register, using ICD-10 code G40. In sensitivity analysis, we required the identified cases of epilepsy to also have prescribed use of antiepileptic drug use ascertained through Anatomical Therapeutic Chemical code N03, according to the Swedish Prescribed Drug Register.

Plants

Seed stocks

Report on the source of all seed stocks or other plant material used. If applicable, state the seed stock centre and catalogue number. If plant specimens were collected from the field, describe the collection location, date and sampling procedures.

Novel plant genotypes

Describe the methods by which all novel plant genotypes were produced. This includes those generated by transgenic approaches, gene editing, chemical/radiation-based mutagenesis and hybridization. For transgenic lines, describe the transformation method, the number of independent lines analyzed and the generation upon which experiments were performed. For gene-edited lines, describe the editor used, the endogenous sequence targeted for editing, the targeting guide RNA sequence (if applicable) and how the editor was applied.

Authentication

Describe any authentication procedures for each seed stock used or novel genotype generated. Describe any experiments used to assess the effect of a mutation and, where applicable, how potential secondary effects (e.g. second site T-DNA insertions, mosiacism, off-target gene editing) were examined.
